# The Influence of Surface Modification with Biopolymers on the Structure of Melt-Blown and Spun-Bonded Poly(lactic acid) Nonwovens

**DOI:** 10.3390/ma15207097

**Published:** 2022-10-12

**Authors:** Ewelina K. Pabjańczyk-Wlazło, Adam K. Puszkarz, Anna Bednarowicz, Nina Tarzyńska, Sławomir Sztajnowski

**Affiliations:** Faculty of Material Technologies and Textile Design, Institute of Material Science of Textiles and Polymer Composites, Lodz University of Technology, 116 Żeromskiego Street, 90-924 Lodz, Poland

**Keywords:** materials, biopolymers, layers, nonwovens, PLA, melt-blown, spun-bond, electrophoretic deposition, sodium alginate, sodium hyaluronate, micro-CT

## Abstract

The article presents the continuation of the research on modification of fibrous carriers based on poly(lactic acid) using the electrophoretic deposition (EPD) method by the two types of biocompatible polymers—sodium hyaluronate and sodium alginate. Such modified nonwovens, differing in the structural parameters due to different manufacturing methods, could be potentially used in different biomedical applications. The results of the analysis indicate that the EPD process significantly changes the structural characteristics of the carrier in terms of thickness and porosity, which not always can be beneficial in terms of the final application. The varying structure of both carriers significantly influences the mode of deposition of the layer, the efficiency of the deposition process as well as the structural characteristics of the carrier after deposition. Microtomographic and SEM studies were employed to analyze the structure of deposits, and FTIR analysis allowed for confirmation of the occurrence of the polymer layers and its chemical structure.

## 1. Introduction

The need for a responsible approach to the management of natural resources, environmental protection and sustainable development resulted in an intensive search for new materials that would meet the requirements of ecology but would not differ in their characteristics from commonly available materials. New materials are produced and utilized in the construction, aviation, automotive and space industries, the production of various goods, but also in the broadly understood sector of multi-purpose textiles, by which we mean not only clothing textiles, but also utility textiles (e.g. technical, special-purpose textiles, smart textiles), as well as various types of protective and medical textiles.

In the framework of our research, we attempted to modify nonwovens, which could potentially be used in biomedical application, with two types of natural polymers. The aim of this was to check the possibility of modifying the surface of the nonwoven with biopolymers, which in further steps of research development could give virtually unlimited possibilities of enriching the layer with various types of active substances. The research part presented in this article provides the continuation of studies undertaken in the field of polymer modification of fibrous structures, as presented elsewhere [1], and is focused on the determination of the influence of polymeric surface modification on the structure of two types of nonwovens, differing in their structural properties as a result of being produced by two different methods—spun-bonding and melt-blowing processes.

In our study, the carrier was made of poly(lactic acid) (PLA), which is a polyester of lactic acid or 2-hydroxy propionic acid [2]. PLA can be obtained from lactic acid by two methods: ring opening polymerization and direct polycondensation [3,4,5,6]. It exhibits good mechanical properties and is entirely biodegradable and biocompatible. However, the polymer exhibits some disadvantages that reduce its use in many applications—brittleness and slow crystallization [2,7]. The properties of the polymer are highly correlated with its semicrystalline morphology, implying that they depend on the degree of crystallinity and crystal structure [8,9]. PLA is also broadly used in medicine. It can be applied as a drug carrier for active pharmaceutical ingredient, such as a protein or peptide, being homogeneously distributed in the polymer matrix [10,11]. Furthermore, the properties of PLA enable rapid prototyping and efficient creation of structures through 3D printing—this allows manufacturing personal protective equipment or personalized tissue scaffolds [12,13]. In addition, it is also often employed in tissue engineering and regenerative medicine. Among many others, it can support tissue regrowth in bone grafting procedures [12]. PLA is also applied in tissue regenerative medicine, as poly(lactic acid)-based products may contain bioactive substances that promote wound healing [14,15]. PLA nonwovens can also be modified by other methods; Wang et al. [16] used magnetron sputtering to deposit nanostructured silver films on nonwoven surfaces for application in wound dressings. Superhydrophobic and superoleophilic PLA nonwoven fabric with stereocomplex (SC) crystals have also been manufactured by a nonsolvent-induced phase separation method using only a small amount of poly(D-lactic acid) (PDLA) [17] and modified Ti(OBu)_4_ and HFA [18] for oil/water separation purposes. Furthermore, antibacterial hybrid materials consisting of nonwoven poly(lactide) and incorporating the organophosphorus compound phosphomycin as a coating and modifying agent [19] can also be obtained.

In our study, for coating purposes two types of polymers were used—sodium hyaluronate and sodium alginate. Sodium hyaluronate is the sodium salt of hyaluronic acid and a high molecular weight biopolysaccharide [20]. It is also a naturally occurring extracellular matrix glycosamine-glycan that has an important role in wound healing and inflammation [21,22,23,24,25]. It exhibits biocompatibility and good water sorption [26]. Due to its properties, it is widely used in drug delivery systems [27,28]. Furthermore, it may be used in the diagnosis and treatment of bladder cancer [29]. Sodium alginate is a naturally occurring polysaccharide found both in algae and soil bacteria [30]. It finds application in many industrial fields—food, medical, pharmaceutical or textile—due to its gel-forming properties and ability to retain water. Moreover, it presents biocompatibility, biodegradability and chelating properties [31]. All these features have made it an excellent material for drug delivery systems [32,33] and tissue engineering [33,34].

Nowadays, the textile industry is focused on producing textiles with innovative, multifunctional properties. Self-cleaning, UV-protective and antibacterial products are being developed. These modifications are mainly based in surface modification by applying different kinds of layer on the fibrous carriers and apart from increasing functionality, ought to be economical and environmentally friendly. One such modification is based on sodium alginate and titanium dioxide nanoparticles. Application of these substances onto polyester fabric results in acquiring outstanding antibacterial properties towards the Gram-negative bacterium *E. coli*, which persisted after five washing cycles [35]. Polyester and polyamide fabrics modified with sodium alginate, which were subsequently treated with copper ions, have also been investigated. The modified polyester fabric showed superb antimicrobial activity against Gram-negative *E. coli*, Gram-positive bacteria *S. aureus* and yeast *C. albicans*, whereas polyamide exhibited maximum antibacterial and fungistatic activity [36]. Antimicrobial modification of textiles through the use of chitosan is also possible [37,38]. Most commonly, it is performed on cotton, however, it is also possible on polypropylene nonwovens. Chitosan is covalently bonded to polypropylene with carboxylic acid using carbodiimide dissolved in water—the obtained material is characterized by improved wettability and antimicrobial properties against *P. aeruginosa* [39]. Antimicrobial properties of polypropylene nonwoven fabric modified with chitosan oligomers were also examined and found to reduce by over 90% the activity of *P. vulgaris*, *S. aureus* and *E. coli* [40]. 

Similarly, sodium hyaluronate is another biopolymer used to modify textiles. Viscose fabric was modified with a natural antimicrobial cationic surfactant derived from lysine and hyaluronic acid. The material revealed antimicrobial activity against *E. coli*, *S. aureus*, *S. agalactiae*, *C. albicans* and *C. glabrata* [41]. Textiles containing polymeric coatings supporting wound healing are also being obtained [42]. Among these is coating of a polypropylene nonwoven fabric with a layer of dibutyrylchitin, which is a soluble derivative of chitin [43]. Fibers and nonwovens made of dibutyrylchitin have also been produced and have shown satisfactory healing results in both burn and postoperative wounds [44]. Sodium hyaluronate is another polymer out of which nonwovens are made. Such dressings have demonstrated non-cytotoxicity and non-pyrogenicity, which combined with the properties of sodium hyaluronate—biocompatibility and non-cytocompatibility—make them highly promising for wound healing applications. Lin et al. [45] examined the effect of a hyaluronic acid coating in improving the biocompatibility of PGA. In vitro characterization showed that the HA coating can enhance cell adhesion to the scaffold and reduce gene expression. Dressings with antimicrobial properties were also manufactured from cotton nonwoven fabric modified using a layer-by-layer technique with sodium hyaluronate and chitosan [46]. Another polymer used to produce dressings is chitosan [47,48]. Montaser et al. [49] developed a dressing for burn wound healing. Silver nanoparticles were produced on a catonized cotton gauze fabric into which the drug was incorporated, followed by application of a chitosan solution to the product. Textile scaffolds have as well been produced to promote cell growth. Risbud et al. [50,51] coated a polyester fabric with a biodegradable chitosan-collagen membrane. Such a scaffold has a three-dimensional structure and, therefore, a larger surface area for cell adhesion and growth. Research was also conducted to create a scaffold from palmitoyl-hyaluronate, and wet-formed fibers were coated with fibronectin, fibrinogen, laminin and collagen. The best cell growth was reported on material coated with fibronectin and fibrinogen [52]. Similarly, a scaffold made of poly ε-caprolactone reinforced with gelatin from calf skin can be produced. Such a structure affects biological processes related to nerve regeneration by enhancing cell adhesion, proliferation and differentiation [53].

The article presents research on polymeric modification of fibrous carriers based on PLA, differing with structural characteristics, which are the consequence of the manufacturing process by the two types of biocompatible polymers—sodium hyaluronate and sodium alginate. The emphasis of the study was placed on the structural changes that occur after the EPD process, with focus on the possible deposition modes and influence on the selected structural parameters of the carriers after deposition.

## 2. Materials and Methods

### 2.1. Materials

The following polymers were used in the study: sodium alginate (SA; Protanal^®^ LF 10/60 L Alginate, Mv = 89 Da) and sodium hyaluronate (SH; Mv = 1.8–2.0 MDa), purchased in FMC Biopolymer Corporation (USA/Europe, Brussels, Belgium) and Contipro Biotech (Dolni Dobrouc, Czechia), respectively. The solutions of 1.5% of SA and SH were prepared using distilled water and mechanical stirring. The electrodeposition was carried out in the previously selected conditions: U = 30 V and U = 35 V, T = 25 °C and t = 3 min, as described in more detail in [1]. The samples were dried at room temperature until they reached a constant mass. 

The subject of the research were two nonwovens made with polylactide (PLA) using two different processes: melt-blowing and spun-bonding. Both types of nonwovens are often used for medical purposes, as standard medical textiles, e.g., liners, wound dressings, disposable materials and others. The poly(lactic acid)—PLA in the form of granules of density 1.24 g/cm³—was purchased in Cargill (Natureworks, Ingeo^TM^ 6201D, Plymouth, MN, USA). The PLA nonwoven carriers were characterized in terms of surface mass in accordance with the PN-EN 29073-1:1994 standard [54]. The thickness of the nonwovens was determined according to X-ray micro-CT analysis. The characteristics of the tested textiles are presented in Table 1. 

Micro-CT is one of the emerging and most accurate cross-sectional imaging techniques for spatial objects providing 3D images data using tomographic reconstruction. In particular, 3D imaging appears to be of greatest importance for textile products and fibrous materials, the structure of which differs significantly from compact polymer blocks, ceramics and metal materials. Very interesting works on the subject of 3D imaging using microtomography of various types of fibrous structures are carried out by K. Schladitz [56] and P. Verma et al., who studied the induction of auxetic and out-of-plane auxetic response in needle-punched nonwovens [57,58]. 

**Figure 1:**
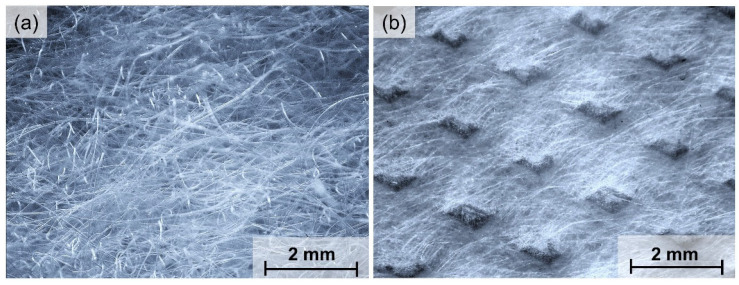
Optical microscopy images surface of tested nonwovens based on PLA: (**a**) melt-blown; (**b**) spun-bonded.

Optical microscopy images of tested nonwovens using optical microscope Delta Optical Smart 5MP PRO (Delta Optical, Warsaw, Poland) and software Delta Optical Smart Analysis Pro 1.0.0 were presented in Figure 1.

On the basis of the presented images, it can be observed that the melt-blown nonwoven fabric is characterized by a chaotic spatial distribution of folded fibers, while spatial arrangement of fibers in spun-bonded nonwoven fabric is more ordered, and on its surface a periodic pattern is visible, created in the manufacturing process at the calendering stage.

### 2.2. Methods

#### 2.2.1. Nonwovens Fabrication

**Figure 2:**
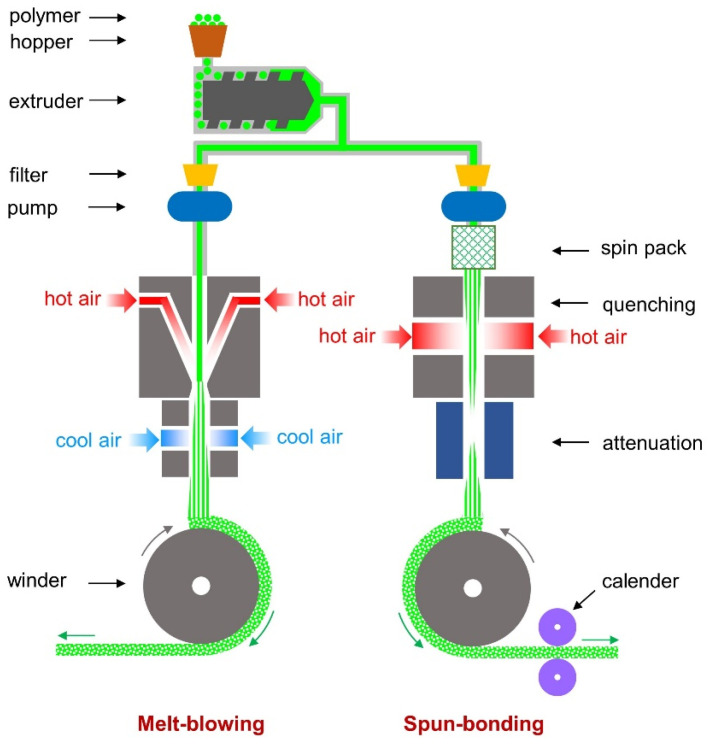
Comparison of two applied techniques of PLA nonwoven fabrics production.

Both the melt-blown and spun-bonded methods are successfully used in the production of various types of textiles, including medical-grade textiles, mainly single-use. Both processes are schematically presented in Figure 2.

Melt-blown (MB) and spun-bond (SB) nonwovens are composed of filaments produced by extruding a polymer through ultra-fine holes. Once the fibers are formed, they are drawn out and then collected to form a nonwoven fabric. The major difference between the melt-blowing and spun-bonding processes occurs during the drawing out phase. In the melt-blowing process, after forming, fibers are subjected to a high-velocity stream of hot air to obtain fibers with a smaller diameter [59]. Therefore, the thickness of fibers obtained through the SB method is 15–40 µm, whereas the MB method is 2–10 µm [60]. Flexibility is considered an asset of both types of nonwovens, however, only SB nonwovens are characterized by relatively high mechanical strength. On the contrary, MB nonwovens are often found in composite materials (mainly SB/MB/SB) [61,62]. Whereas, the drawbacks are the inability to blend different types of polymers and to obtain fibers with significantly different diameters [63,64]. Both MB and SB nonwovens are employed successfully as tissue scaffolds [65,66,67]. Selection of the adequate structure is determined by the future application of the product, and the mechanical properties must be selected appropriately for the forces occurring during tissue growth [68]. SB nonwovens are characterized by the presence of many regular small pores in their structure, whereas in the structure of MB nonwovens, small diameter pores can be found; however, they are very irregular [69]. Studies show that both MB and SB nonwovens made of PLA, compared to electrospun and carded nonwovens, result in a significant increase in cell proliferation within the first 7 days of culture [70]. The conditions of the melt-blown process were as follows: a temperature range of the process—200–220 °C— and the polymer throughput rate—30 Nm^3^/h. For spun-bond: a temperature in the range from 205–216 °C; a polymer throughput rate in the range of 0.10–0.43 g/min. The calender temperature was varied from 60–130 °C. 

#### 2.2.2. Electrodeposition

**Figure 3:**
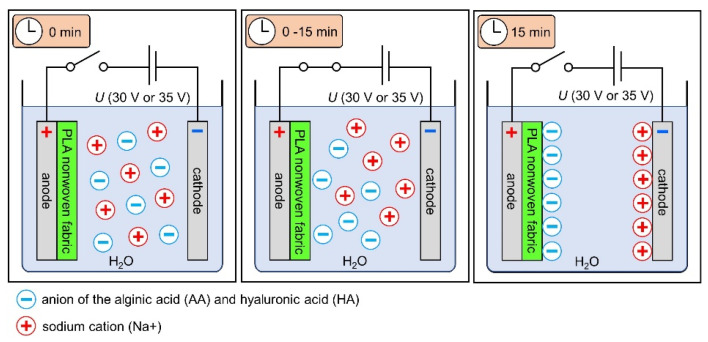
Scheme of surface modification of tested nonwovens. The figure was sourced and modified based on [1].

Electrophoretic deposition, in brief, is based in depositing layers of different chemicals on the electrode or a carrier covering the electrode, which can be, among others, metallic or polymeric. In chemical language, the deposition refers to the motion of particles or macromolecules towards the electrode under the electric field, which results in the deposition of their layer on the electrode or a carrier. Electrophoretic deposition (EPD) allows for control over the thickness and morphology of the obtained layers by adjustments in deposition solution characteristics, including type of the polymer and its concentration, as well as process parameters (e.g., voltage, deposition time). An in-depth study of the effects of various parameters on electrodeposition has been presented elsewhere [1]. The applied optimal process parameters of the EPD process were selected in the preliminary studies. The process voltage was 30 and 35 V; EPD was conducted for 3 min at T = 25 °C. The samples were dried at room temperature. In order to select samples for structural studies, samples from a test series conducted for 5 independent experiments were selected. The samples were sized according to the surface area of the electrodes to which they were attached (5 × 5 cm). Representative samples were selected for the study, which showed uniformity of deposition in the visual assessment and allowed to perform FTIR, SEM and micro-CT examinations. The schematic representation of the EPD process with the use of the textile as a carrier is presented in Figure 3. 

#### 2.2.3. Evaluation of the Modified Nonwovens’ Structural Properties

##### X-ray Micro-Computed Tomography (Micro-CT)

Structural parameters (Table 1 and Table 2) and 3D reconstruction of the tested nonwovens were determined using X-ray micro-computed tomography (SkyScan 1272; Bruker, Kontich, Belgium). Micro-CT outcomes were obtained applying the following scanning conditions: X-ray source voltage 50 kV, X-ray source current 200 µA and pixel size 5.5 µm. A 180° rotation was performed with a rotation step of 0.2° and no filter was selected. Micro-CT is based on the absorption of X-rays by the tested material and allows to characterize the internal structure of objects in the microscale. When the tested material consists of phases with different absorption of X-ray radiation (e.g., pores or other spaces and parts constituting of the material), it is possible to identify these phases, determine its shape, volume, surface and spatial orientation inside the material and thus calculate the porosity and determine the characteristics of the pore structure. In such way, in the presented research, it was possible to determine the porosity of nonwovens (air content in the nonwoven fabric) and the content of material deposited in the EPD process using tomography. Moreover, the material deposited on the nonwovens was visualized (green color) on the three-dimensional reconstructions. In order to finally confirm the influence of the modification on the structure of both tested nonwovens, the presented results of tomography were based on the analysis of modified nonwovens in 5 independent experiments (in identical selected four EPD parameters). The geometric parameters of the tested textiles (thickness, total porosity, volume of the nonwoven occupied by the deposited material and deposited layer thickness) presented in Table 1 and Table 2 are the average values of measurement of five separate samples of a given type, while the most representative fragments of samples were selected for the three-dimensional reconstructions of unmodified and modified nonwovens.

##### Fourier Transform INFRARED SPECTROSCOPY (FTIR)

**Figure 4:**
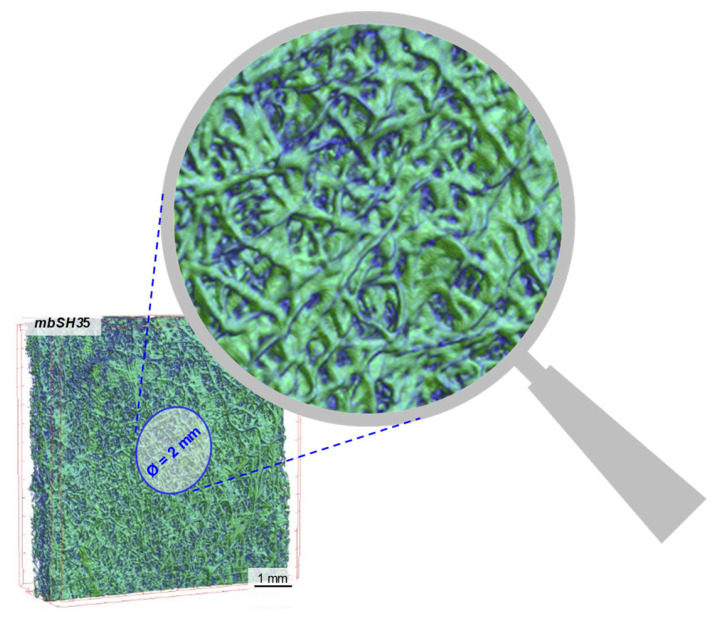
FTIR analysis area (2-millimeter diameter circle).

The study with the use of the IR reflection absorption spectroscopy method was aimed at determining the change in the chemical structure of the surface of nonwoven samples and identification and confirmation of the resulting coating. The FTIR-ATR reflection method was used, with the Thermo Scientific NICOLET 6700 device and the Smart iTR adapter with a Dia crystal with a reflection angle of 45° (Waltham, MA, USA). The spectra were recorded in the wavenumber range 600–4000 cm^−1^ with resolution 4 cm^−1^ in the relation A = f (1/λ). A total of 32 spectra were recorded in the marked range. The OMNIC 8.0 program (Waltham, MA, USA) was used to analyze the spectrograms and determine the characteristic bands in the 700–3800 cm^−1^ range. The Hummel Defined Polymers IR spectra library was used for analysis of the spectrum. The FTIR analysis area of the modified nonwoven is schematically presented in Figure 4.

##### Microscopic Analysis

SEM images were taken with a high-resolution scanning electron microscope—FEI Nova NanoSEM 230 (Jeol, Peabody, MA, USA)—equipped with an electron gun with field emission (FEG). The studies were carried out using low vacuum environment and beam energy in the scope of 10 keV to 15 keV, detector voltage 10 kV and spot 3 kV. The surface-sensitive quantitative spectroscopic technique such as X-ray photoelectron spectroscopy (XPS) was used to analyze the elemental composition of the carriers. The EDX analysis was obtained by scanning with an electron beam of the surface of the material under examination and then monitoring by EDX of the emitted energy lines of the characteristic X-ray radiation of the elements studied.

## 3. Results

### 3.1. Micro-CT Analysis

**Figure 5:**
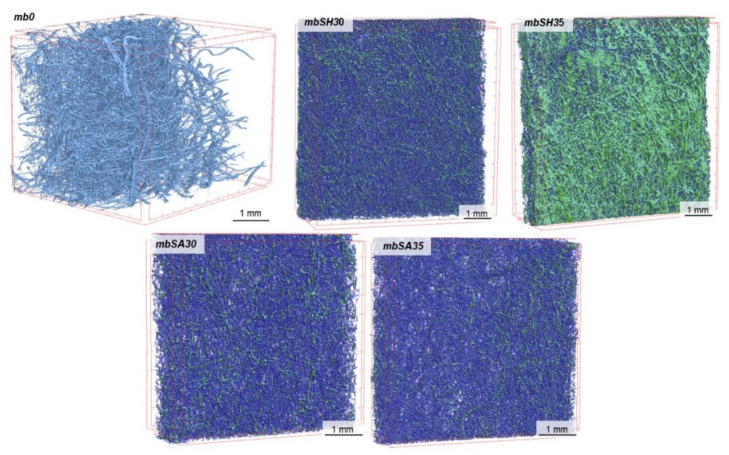
Micro-CT images of unmodified and modified melt-blown PLA nonwoven fabrics.

**Figure 6:**
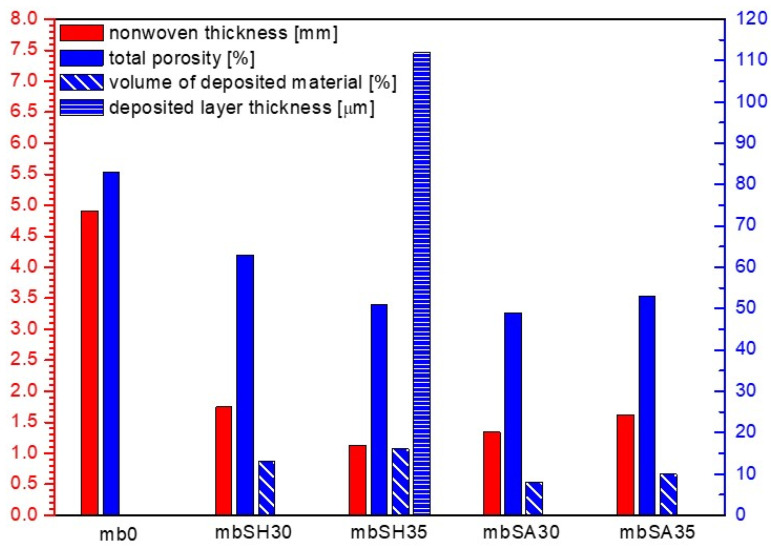
Structural parameters of unmodified and modified melt-blown PLA nonwoven fabrics.

**Figure 7:**
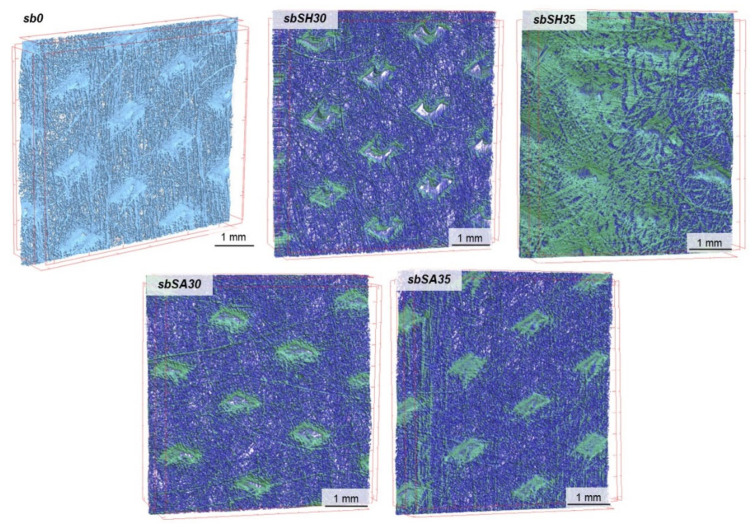
Micro-CT images of unmodified and modified spun-bonded PLA nonwoven fabrics.

**Figure 8:**
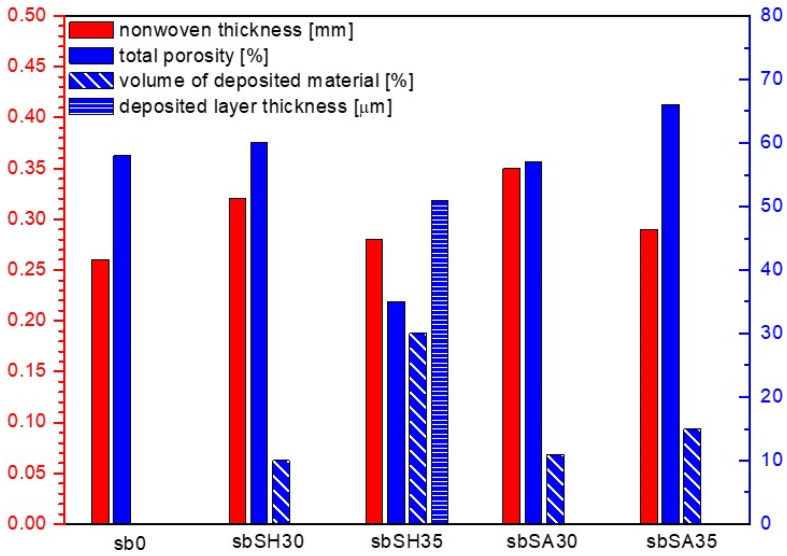
Structural parameters of unmodified and modified spun-bonded PLA nonwoven fabrics.

The characteristics of the tested nonwovens in terms of thickness, total porosity, surface mass, volume of the nonwoven occupied by the deposited material and deposited layer thickness are presented in Table 2. The results of the microtomographic analysis and comparison of structural characteristics are presented in Figure 5, Figure 6, Figure 7 and Figure 8.

Based on the above analysis, it was possible to observe that the EPD process significantly reduces the thickness of the modified melt-blown nonwovens as compared to the unmodified nonwovens and in contrast to spun-bonded nonwovens, in which thickness increased for samples prepared in the process voltage of 30V. For MB nonwovens, the following thickness loss in reference to the unmodified sample was observed: mbSH30 (−64%), mbSH35 (−77%), mbSA30 (−73%), mbSA35 (−67%), while for SB nonwovens, the following thickness change was measured: sbSH30 (+23%), sbSH35 (+8%), sbSA30 (+35%), sbSA35 (+12%). The reduction in thickness in the case of MB nonwovens could be explained by the process of the compression of the very loose structure (Figure 5) and sticking of fibers due to the specificity of the process (immersion in the solution). While, the decrease in thickness for SB nonwovens obtained in higher voltage might also be a result of the compression due to an increase in motion of polymers particles and higher efficiency of the deposition, as shown in [1].

The electrodeposition process also influenced the total porosity of both types of nonwovens. For melt-blown nonwovens, the general decreasing trend after deposition and following total porosity loss were observed: mbSH30 (−24%), mbSH35 (−39%), mbSA30 (−41%), mbSA35 (−36%), while for spun-bonded nonwovens, the following total porosity did not present unequivocal trends and the following changes were observed: sbSH30 (+3%), sbSH35 (−40%), sbSA30(−2%), sbSA35 (+14%). These differences might result from the heterogeneity of the carriers, which is an inherent effect of the process of obtaining them and the averaging of the results obtained from different (representative) measurement sites used for the analysis. Such unpredictable porosity results for spun-bonded can also be caused by the differences in the electrical charge distribution along the surface of the spun-bonded nonwoven and the way in which the deposit is oriented, which is discussed in the conclusion section. Those differences affect the direction of the charged molecules’ motions and might cause uneven distribution of the deposit (e.g., in the form of islands).

The EPD process for both types of nonwovens resulted in a continuous layer only in the case of sodium hyaluronate and deposition at 35V (samples: mbSH35 and sbSH35), which could also be a result of the heterogeneity of the carrier structure. The thickness of the deposited layer was 112 μm for the melt-blown nonwoven and 51 μm for the spun-bonded nonwoven. For the remaining modified nonwovens (mbSH30, mbSA30, mbSA35, sbSH30, sbSA30, sbSA30) the deposited occurred to be distributed along the surface in the form of islands. Similarly, in the case of the spun-bonded nonwoven, a continuous coating was obtained only with the sample sbSH35, which had the lowest porosity and almost the smallest thickness. In the case of melt-blown nonwovens, the largest fraction of the volume (16%) was observed for mbSH35, while in the case of spun-bonded nonwovens the largest fraction was found for sbSH35 (30%). 

### 3.2. FTIR Analysis

**Figure 9:**
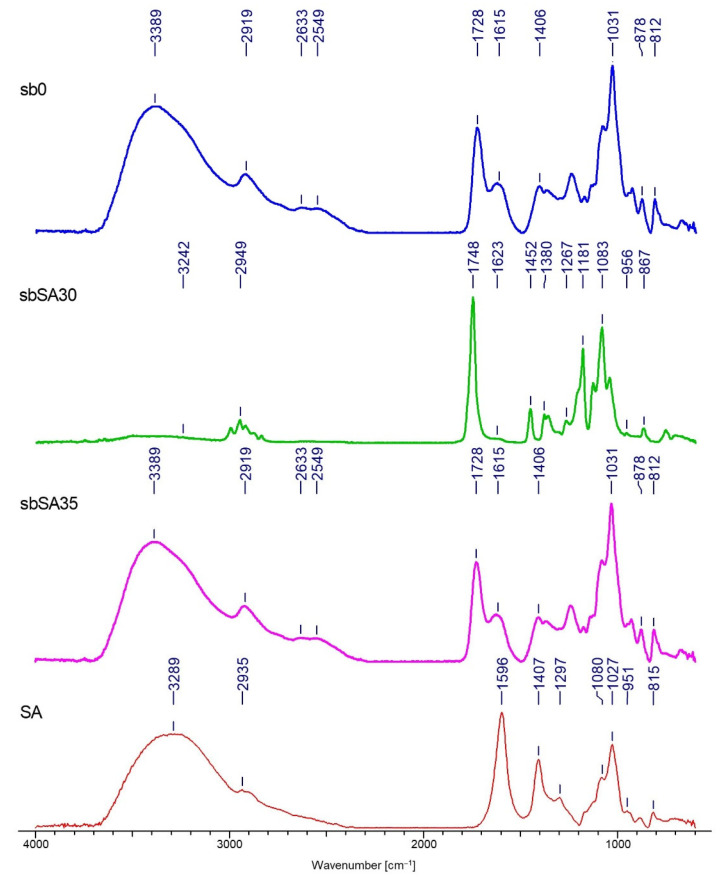
FTIR spectra of the spun-bonded PLA nonwoven fabrics with deposition of the sodium alginate layer (SA): sb0—reference sample (spun-bonded PLA nonwoven); SA—powder of sodium alginate.

**Figure 10:**
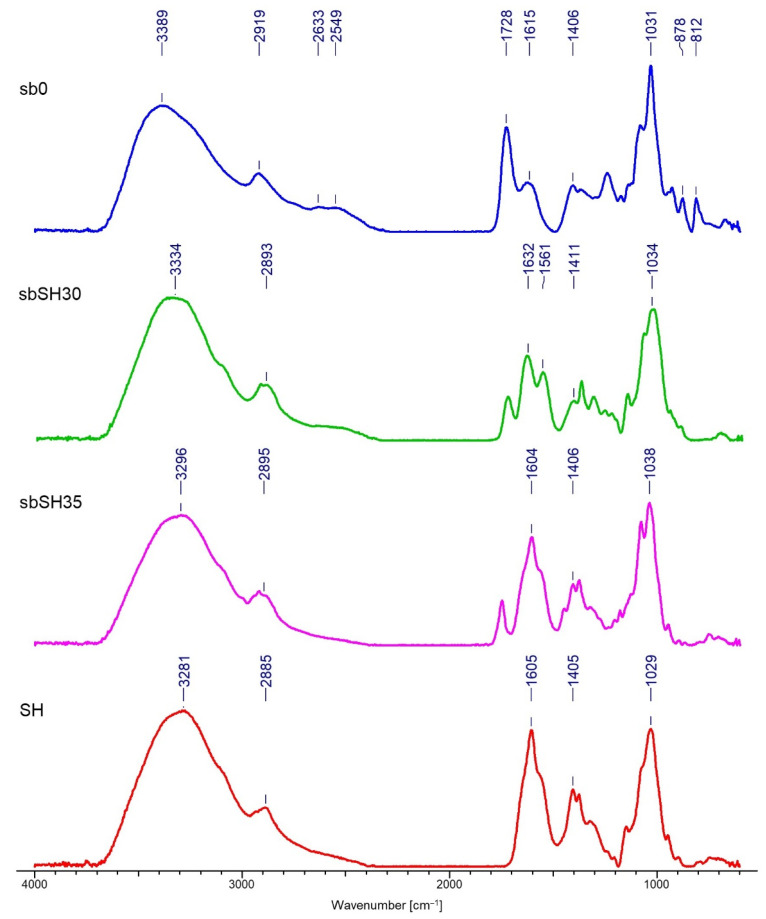
FTIR spectra of the spun-bonded PLA nonwoven fabrics with deposition of the sodium hyaluronate layer (SH): sb0—reference sample (spun-bonded PLA nonwoven); SH—powder of sodium hyaluronate.

**Figure 11:**
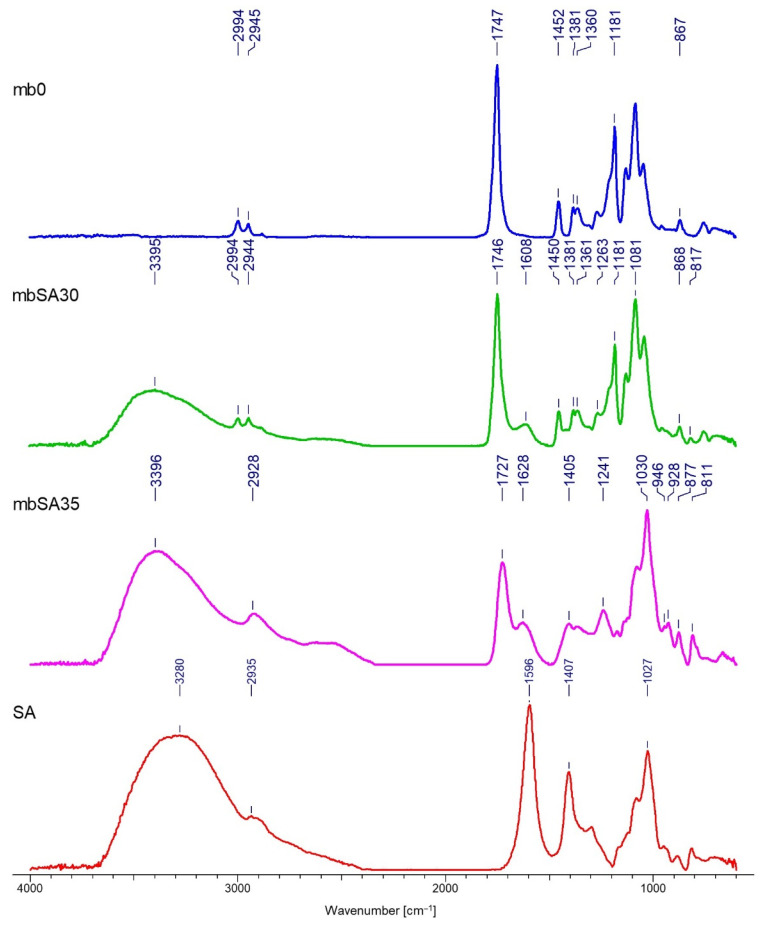
FTIR spectra of the melt-blown PLA nonwoven fabrics with deposition of the sodium alginate layer (SA): mb0—reference sample (melt-blown PLA nonwoven); SA—powder of sodium alginate.

**Figure 12:**
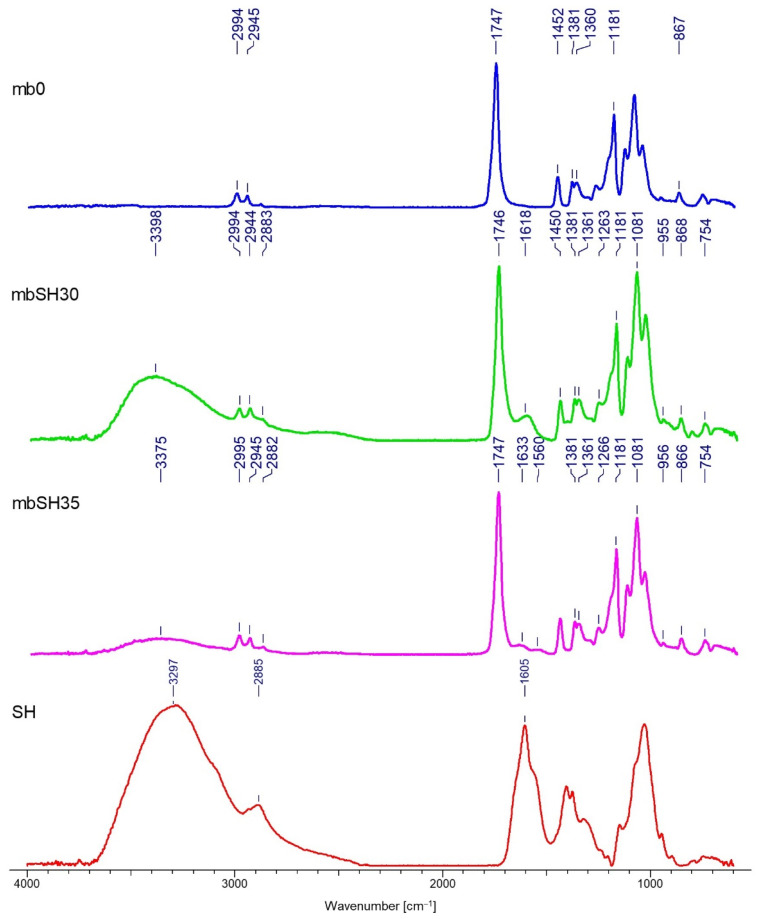
FTIR spectra of the melt-blown PLA nonwoven fabrics with deposition of the sodium hyaluronate layer (SH): mb0—reference sample (melt-blown PLA nonwoven); SA—powder of sodium hyaluronate.

Spectrograms characterizing the chemical structure of the tested samples are presented in Figure 9, Figure 10, Figure 11 and Figure 12.

The FTIR analysis confirmed the deposition of both polymers on two types of nonwovens obtained by different techniques. The results presented in Figure 9, Figure 10, Figure 11 and Figure 12 allow for the conclusion that the PLA nonwoven material after the EPD process shows different efficiency of the process and the degree of deposition for two types of the applied polymers—SH and SA. In both cases, two phenomena are observed: the appearance of new absorption peaks characteristic for polysaccharides in general: 

Sodium alginate—the band at 3289 cm^−1^, which can be assigned to -OH groups, which is not seen in the poly(lactic acid)/reference sample (without deposit) and at 1596 cm^−1^, which can be attributed to the asymmetric stretching of O-C-O groups. The weak signal at 2935 cm^−1^ can be assigned to C-H stretching vibrations, and the band at 1407 cm^−1^ can be assigned to C-OH deformation vibrations. The right part of the spectrum presents few weaker bands, and the band at 1027 cm^−1^ can be assigned to C-O and C-C stretching vibrations. The region 950–750 cm^−1^ applies to uronic and mannuronic acid residues. The peak at 3280 cm^−1^ is less intensive for the samples with deposits than for SA and it does not occur in the reference sample, which confirms that deposition took place;

Sodium hyaluronate—these are bands at 3297 cm^−1^ and 1605 cm^−1^, which can be attributed as in the case of sodium alginate. The band at 3297 cm^−1^ (a) can be assigned to -OH groups and is absent in the poly(lactic acid)/reference sample. The band at 2885 cm−1 can be assigned to -NH stretching band;

The increase in the intensity of the above characteristic peaks together with the increase in the process voltage from 30 to 35V. In general, for both polymers, by increasing the process voltage, it was possible to increase the deposition rate, which is especially evident with spun-bond nonwoven. Only in the case of one sample of MB nonwoven (Figure 12) this trend did not occur, which might be caused by the heterogeneity of the carrier structure and its impact on the deposition process. 

The FTIR analysis allowed confirmation of the chemical structure of the deposits for both polymers. A detailed spectral analysis of the deposits based on sodium hyaluronate and sodium alginate on PLA fibrous structures obtained by electrodeposition and their characteristic bands has been presented in previous articles of the research group [1].

### 3.3. Microscopic Analysis 

**Figure 13:**
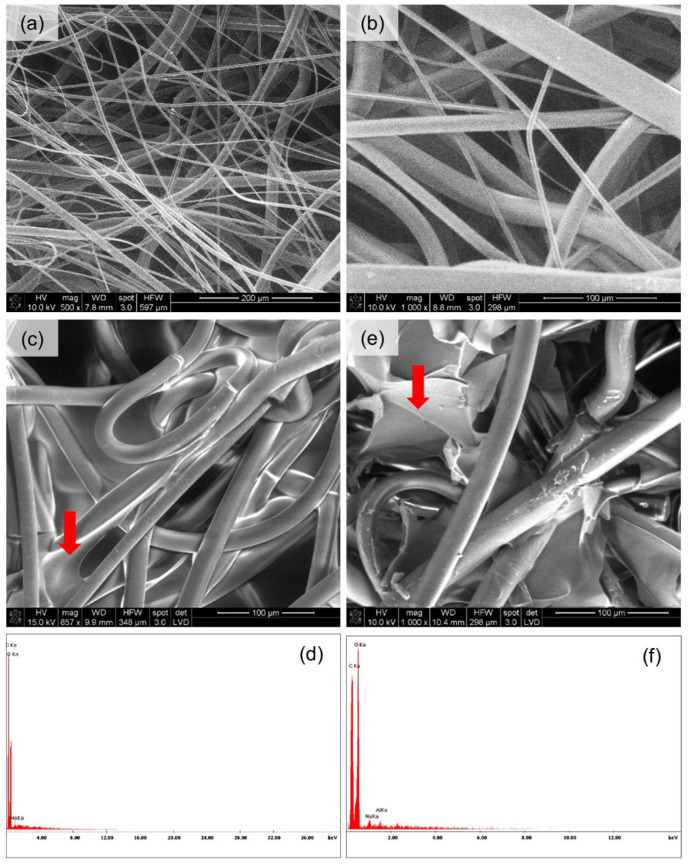
SEM and EDX images of the melt-blown PLA nonwoven fabrics: (**a**,**b**) reference sample—melt-blown PLA nonwoven without deposit; (**c**,**d**) sample with sodium alginate deposition (3 min, 35 V, T = 25 °C); (**e**,**f**) sample with sodium hyaluronate deposition (3 min, 35 V, T = 25 °C).

**Figure 14:**
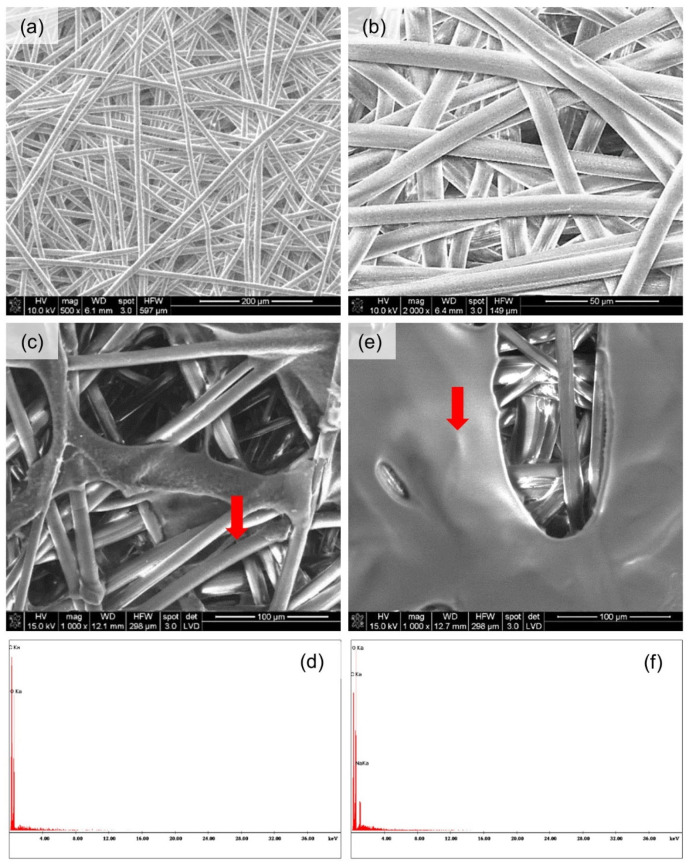
SEM and EDX images of the spun-bonded PLA nonwoven fabrics: (**a**,**b**) reference sample—spun-bonded PLA nonwoven without deposit; (**c**,**d**) sample with sodium alginate deposition (3 min, 35 V, T = 25 °C); (**e**,**f**) sample with sodium hyaluronate deposition (3 min, 35 V, T = 25 °C).

Figure 13 and Figure 14 present the spatial analysis of the deposits carried out with an SEM microscope with EDX analysis.

The SEM analysis in both cases of nonwovens revealed the previously discussed heterogeneous structure of the carriers. In the case of melt-blown nonwovens (Figure 13a), the fibers are loosely and unevenly distributed, without the selected direction of spatial orientation, have a different diameter and places with higher and lower fiber density can be observed. In the case of spun-bonded nonwovens (Figure 14a), the individual fibers also do not have a specific orientation in space; however, it should be noted that the density of the fibers appears more uniform, and the fibers themselves have a similar thickness. The EDX analysis showed the presence of a large number of oxygen and carbon atoms both from PLA and deposits and a sodium atom which comes from the polymers (sodium salts of hyaluronic acid and alginic acid were used in the experiment). In one case (Figure 14b), the elemental analysis showed no sodium atoms, where probably the wrong measurement site was chosen. In the case of the melt-blown technique, the following observations were taken: the reduction in thickness of the nonwoven fabric is associated with massive sticking of the fibers, and the occurrence of the compression effect under the influence of water. This relationship is more emphasized for melt-blown nonwoven than spun-bonded, due to the large differences in their structures. Melt-blown nonwovens exhibit much looser structure and orientation of the fibers and initial greater thickness. While for spun-bonded nonwovens, besides the visible sticking of the fibers, the effect of an increase in thickness after deposition is noticeable, which may result from creation of a polymer layer on the surface of the nonwoven. When assessing the homogeneity of the polymer layer, it should be noted that in both cases the presence of the layer is visible; however, in the case of the melt-blown nonwovens, this layer does not cover the entire surface of the carrier but is distributed randomly throughout the entire volume of the nonwoven. In the case of spun-bonded nonwovens, we can observe both—areas covered with a relatively dense polymer layer (Figure 14c) and areas where this layer is practically absent or only occurs locally (Figure 14b). The reason for this may be the phenomenon of uneven distribution of electric charges related to the structure of calenders, which affect the accumulation of charges around calenders, which could change the course of movement of the charged polymer particles and pull them towards places with accumulated charges. In all analyzed samples, a layer of deposit can be observed, the same as significant phenomena of nonwoven compression and reorientation of fiber filaments due to sticking. 

## 4. Discussion

The research aimed at verifying whether and how layers of biopolymers can be deposited on the surface of fibrous carriers. It was assumed that biopolymer coating will influence the structural characteristics of the nonwoven carriers and the objective was to answer to what extent. The analysis confirmed that the electrophoretic deposition with biopolymers is possible, and it might bring interesting and promising possibilities of surface modification of various types of nonwovens by means of a simple method such as EPD. Based on the conducted analysis, and as expected, it significantly influences the structure of the nonwoven carriers. The average thickness loss in reference to the unmodified sample for the melt-blown nonwoven (and both polymers) amounted to 70.25%. However, having the same deposition time and different process voltage, no linear tendency was noticed. In the case of the mbSH30 sample, the increase in voltage by 5 V caused the decrease in thickness of the layer; while for the mbSA30 sample, the same increase in voltage resulted in a thickness increase of almost 0.3 mm. In this case, the change of 5V in the applied voltage of the process resulted in the decrease in the thickness of the carrier of 12.5% for samples with sodium hyaluronate and 17.14% for samples with sodium alginate. This effect of the reduction in thickness of the fibrous carriers might be due to the compression and sticking of fibers under immersion in water, contact with aqueous polymer solutions and during drying process. Interestingly, the electrophoretic deposition with biopolymers causes, in contrast to melt-blown nonwovens, a significant increase in thickness of the modified spun-bonded nonwovens as compared to the unmodified SP nonwovens. In this case, the average increase in thickness amounted to 19.5%. 

It was noticeably evident that EPD results in changes in thickness and other structural parameters. The electrodeposition process influenced the total porosity of both types of nonwovens. The average total porosity loss for melt-blown nonwovens amounted to 35%, while for spun-bonded, no clear tendency was observed. The presented changes may suggest that the deposition on melt-blown nonwovens causes significant structural changes throughout the whole carrier (not only on the surface), while for spun-bonded, the changes in the structures are less emphasized and are limited to the outer layer of the carrier. For both polymers in the case of melt-blown nonwovens, and the applied voltage of 30 V, the porosity decreased by an average of 33.5%, and for the voltage of 35 V—by 37.5%, which is relatively high. The situation is different for spun-bonded nonwovens which are already partially compressed during the calendering process, which affects the accessibility of the polymer solution to the material itself. Thus, data for spun-bonded nonwovens do not allow to draw an unequivocal conclusion. 

In order to measure and assess the uniformity of the polymer deposition on fibrous carriers, the continuity of the deposit layer was assessed. The EPD process for melt-blown and spun-bonded nonwovens resulted in a continuous layer only in the case of sodium hyaluronate deposition at 35 V. For the remaining modified samples, the deposited layer occurred to be distributed along the surface in the form of “islands” (local aggregations of deposit spread over the surface of the carrier surrounded by spaces without deposit). In the case of spun-bonded nonwovens, the deposited material was located on the edges of the holes made by the calender (where the electric charge is mostly concentrated). This may suggest that any modification of the fabricated nonwoven structure which results in the concentration of charges in certain areas, may be responsible for the uneven distribution of the coating over the entire surface of the carrier. This phenomenon is related to the attraction of charges and ions from polymers dissolved in the solution and their movement under applied voltage. Depending on the intended application, both types of deposit distribution could be considered as advantageous. If the polymer layer is to act as a wound dressing with moisturizing features, the deposit could be applied in an island form. In the case of sodium hyaluronate it could be beneficial, as it is resorbable in the body and the islands would gradually be resorbed over time, while the remaining places (outside the islands) would ensure gas exchange. However, if the coating is to play a barrier role, then it is expected to occur in the uniform and continuous form. 

Interestingly, a uniform coating was obtained only for the melt-blow specimen, in which the highest thickness decrease and almost the highest porosity decrease were observed. In the case of such a non-uniform structure, only a significant reduction in thickness (compression effect) could allow for producing a continuous coating. Similarly, in the case of the spun-bonded nonwoven, a continuous coating was obtained only with the sample sbSH35, which had the lowest porosity and almost the smallest thickness (after deposition there was only a slight increase in the thickness of the nonwoven fabric which can be attributed to the building up of the coating layer). For both types of modified nonwovens, the volume fraction of the deposited material in relation to the volume of the entire nonwoven was analyzed. In both cases of nonwovens, the volume area occupied by the material increased when the applied voltage was increased from 30 V to 35 V for both polymers—sodium hyaluronate and sodium alginate. For the melt-blown nonwoven, this parameter increased by 3% for sodium hyaluronate and by 2% for sodium alginate. For spun-bonded nonwoven, the increase in the volume occupied by hyaluronan in higher voltage was 20% and for sodium alginate, it was 4%. This phenomenon is associated with an increase in the general efficiency of the deposition process with increasing process voltage. Higher voltage causes an increased movement of ions towards the electrodes and carrier, which results in greater efficiency of building up the polymer layer during fixed time than when using a lower voltage. Apart from the above, no further differences were observed between the different types of nonwovens in terms of the volume occupied by the deposited material. 

The results of the FTIR analysis allow for the conclusion that the PLA nonwoven material after the EPD process shows different efficiency of the process and the degree of deposition of the two types of applied polymers—SH and SA. In both cases, two phenomena are observed: the appearance of new absorption peaks characteristic for polysaccharides in general, and the increase in the intensity of those characteristic peaks associated with the increase in the process voltage. The SEM analysis allowed, together with micro-CT studies, for assessing the occurrence and homogeneity of the polymer layer. In both cases, the presence of the layer is visible; however, in the case of melt-blown nonwovens, this layer does not cover the entire surface of the carrier but is distributed randomly throughout the entire volume of the nonwoven. In the case of spun-bonded nonwovens, we can observe both—areas covered with a relatively dense polymer layer (Figure 14c) and areas where this layer is practically absent or only occurs locally (Figure 14b). The reason for this may be the phenomenon of uneven distribution of electric charges related to the structure of calenders, which affect the accumulation of charges around calenders, which could change the course of movement of the charged polymer particles and pull them towards places with accumulated charges. In all analyzed samples, a layer of deposit can be observed, the same as significant phenomena of nonwoven compression and reorientation of fiber filaments. 

## 5. Conclusions

This article presents the research outcomes concerning the determination of the usefulness of the electrophoretic deposition in application in the modification of the fibrous carriers. Within the framework of the study, the parameters of the carrier structure after deposition were analyzed. The fibrous carriers were obtained by two different manufacturing methods. The consequence of this is their different structural parameters. The aim of the research was to determine how the polymer solutions penetrate the carrier structure, how the deposit settles (on the surface or in the volume of the carrier) and what the deposition mode is. Furthermore, our goal was to determine if polymers such as sodium hyaluronate and sodium alginate are suitable for the use in this method because both polymers are known to have very favorable biological properties. As a result, the applicability of using the EPD method to modify fibrous structures was confirmed and interesting observations were made. Both nonwovens were characterized by high heterogeneity of the structure which significantly influenced the analysis and results. However, in comparison to spun-bonded nonwovens, the heterogeneity of the structure resulted to be dominant for nonwovens obtained by the melt-blown method. As a result of the calendering process step, spun-bonded nonwovens are subjected to compression and partial formation of the calendered structure, which significantly influenced the differences between the deposition on both types of carriers. Deposition on spun-bonded nonwoven proved to be relatively more effective; however, the heterogeneity of the deposits was debatable. An important difference between two types of the carrier is the way the layer is deposited. In the case of the spun-bonded nonwoven, the deposition aggregates mainly on the edges of the sample and in the calender hole-like structures (where the electric charge is most concentrated). Whereas in the case of melt-blown nonwovens, the polymer solution penetrates deeply into the carrier causing very significant changes in its structure (e.g., it reduces its thickness, causes the compression effect or changes its porosity parameters). This fact may not necessarily be beneficial for their applicability because melt-blown nonwovens are used very often in various types of filters or dressings; thus, they should maintain relative high air permeability to enable gas exchange or fluid exchange, e.g., between the wound and environment. The relatively high irregularities of the fibrous structures obtained by both methods had a dominant effect on the deposition characteristics; therefore, the research group may undertake further studies on the electrophoretic deposition of the polymer layer on nonwovens with a more regular structure, such as woven or knitted fabrics. Finally, the article presents research results on the polymeric modification of fibrous carriers based on PLA, differing with structural characteristics, which is the consequence of the manufacturing process by the two types of biocompatible polymers—sodium hyaluronate and sodium alginate. Future works concerning this area include also the EPD with active substances and works on antimicrobial finishing of fibrous carriers.

## Figures and Tables

**Table 1:** Characteristics of tested nonwovens.

Nr	Textile Type	Textile Name	Composition	Thickness ^a^ [mm]	Surface Mass ^b^ [g·m^−^^2^]	Total Porosity ^a^ [%]
1	Melt-blown nonwoven fabric	mb0	Polylactide (PLA)	4.900	169.44	83
2	Spun-bonded nonwoven fabric	sb0	Polylactide (PLA)	0.258	48.5	58

^a^ Determined according to X-ray micro-CT. ^b^ Determined according to PN EN 12127:2000 [55].

**Table 2:** Characteristics of the tested nonwovens.

Nr	Textile Type	Textile Name	Modification Parameters			Thickness ^a^ [mm]	Total Porosity ^a^ [%]	Surface Mass ^b^ [g·m^−2^]	Volume of the Nonwoven Occupied by the Deposited Material ^a^ [%]	Deposited Layer Thickness ^a^ [μm]
			Solution	Voltage [V]	Duration [min]					
1	Melt-blown PLA nonwoven fabric	mb0	none	none	none	4.910	83	169.44	0	none
2		mbSH30	1.5% SH	30	3	1.745	63		13	none
3		mbSH35		35		1.130	51		16	112 *
4		mbSA30	1.5% SA	30		1.340	49		8	none
5		mbSA35		35		1.625	53		10	none
1	Spun-bonded PLA nonwoven fabric	sb0	none	none	none	0.260	58	48.5	0	none
2		sbSH30	1.5% SH	30	3	0.320	60		10	none
3		sbSH35		35		0.280	35		30	51 *
4		sbSA30	1.5% SA	30		0.350	57		11	none
5		sbSA35		35		0.290	66		15	none

^a^ Determined according to X-ray micro-CT. ^b^ Determined according to PN EN 12127:2000 [55]. * The continuous deposited layer was obtained only for two modified nonwovens: mbSH35 and sbSH35.

## Nomenclature

AA; SA	alginic acid; sodium salt of alginic acid, sodium alginate
CT; micro-CT	computed tomography; micro-computed tomography
EPD	electrophoretic deposition
FTIR	Fourier transform infrared spectroscopy
HA; SH	hyaluronic acid; sodium hyaluronate
MB	melt-blown
PLA	poly(lactic acid)
SB	spun-bonded
